# Experimental Investigation and Safety Classification Evaluation of Small Drone Collision with Humans

**DOI:** 10.3390/biomimetics10030157

**Published:** 2025-03-03

**Authors:** Chunyu Bai, Yazhou Guo, Qinghua Qin, Yunlai Zhou, Zhigang Li, Yafeng Wang

**Affiliations:** 1 State Key Laboratory for Strength and Vibration of Mechanical Structures, School of Aerospace Engineering, Xi’an Jiaotong University, Xi’an 710049, China; baichunyu2006@163.com (C.B.); yunlai.zhou@xjtu.edu.cn (Y.Z.); 2 National Key Laboratory of Strength and Structural Integrity, Aircraft Strength Research Institute of China, Xi’an 710065, China; guoyazhou623@163.com (Y.G.); wangyafeng623@sina.com (Y.W.); 3 School of Mechanical Engineering, University of Science and Technology Beijing, Beijing 100083, China; zhigangli@ustb.edu.cn

**Keywords:** small drone, experiment, safety grade, head and neck injury, drop height

## Abstract

The safety of small drones in collision with humans has become a key focus in engineering and research fields. This study presents a vertical drop test platform for collision tests involving three representative drones (Air, Mavic 2, and M200) impacting the head of a Hybrid III dummy from different heights and orientations. The deformation and damage of the drones during various collision scenarios, as well as the dynamic responses of the dummy head and neck, are analyzed. The head injury criterion (HIC), together with 3 ms cumulative acceleration (head acc 3 ms), are used to evaluate head injury, while the shear force, axial force, as well as bending moment are applied to evaluate neck injury. The effects of drone type, drop height, and drone collision position on dummy head and neck injury risk are comprehensively analyzed, as well as the quantitative relations between the head and neck injury metrics, and the drop height for the three typical drones are derived. Via the acquired equations, the head and neck injury risks for the three typical drones involved in this study and other similar drones falling from distinct heights shall be predicted. This study proposes a novel method focusing on classifying the safety grades of drone collision with dummy. The safety grades for these three typical drones are categorized via the drop height. The findings further provide crucial data and analytical methods for establishing drone safety standards.

## 1. Introduction

Drones have undergone extensive advancement in recent years. Light and small drones, with an empty weight of less than 15 kg and a maximum takeoff weight of less than 25 kg, dominate the domestic drone market in both consuming and industrial areas [1], leading to vast applications in personal daily life, logistics, security inspections, mapping, and other fields [2,3].

The operation safety of drones has been gaining more attention due to their integration into urban spaces. Under certain circumstances, the online monitoring of light and small drones pose significant challenges. For instance, their operational space frequently overlaps with human activities, leading to frequent accidents induced by technical failures, human errors, or complex ambient conditions, which can cause collisions with humans. For regulatory agencies and the public, drone collision safety has become a critical issue [4]. Although aviation regulations have been raised in certain countries [5,6,7], these rules do not adequately distinguish the exact relation between different types of drone impacts and classify the associated collision safety grades.

Vertical crashes pose a significant collision risk, particularly to the head and neck regions. Scholars have conducted theoretical analyses, experiments, and numerical simulations to assess injuries caused by drone collisions, aiming to establish relations between drone collision scenarios and human injury. For instance, Low [8] established the expression for calculating the impact energy of a UAV applicable to drop impact conditions based on the basic principle of mechanics, which took into account the drop height of the UAV. Furthermore, according to the relationship between the impact kinetic energy and probability of fatality reported by Henderson [9], the effects of the impact energy and drop height on the probability of injury were analyzed. Radi [10] introduced the Blunt Criterion (BC) into the study of UAV–human body impact and considered the complex shape of the contact area between the UAV and the human body. Serious injury (AIS = 3) was defined as the maximum acceptable injury severity; for example, the value (BC = 1.61) corresponding to a 50% risk of skull fracture was used as the injury threshold. Courharbo [11] established the relationship between the UAV mass and the maximum flight speed and further calculated the impact energy. Based on the criteria related to the impact energy (e.g., BC), the UAV mass threshold of weighing less than 250 g was determined for “harmless” drone. The above studies were based on the collision energy of the UAV when analyzing human injury risk, which only considered the linear kinematics of the UAV, lacking the evaluation of angular kinematics and rotationally induced brain injuries.

Theoretical models developed to predict the risk of injury from drone collisions involve significant simplifications and equivalences, which limit their accuracy. Consequently, crash tests and simulations are essential for studying drone impact injury. For example, Weng et al. established a drone simulation model based on the DJI Phantom 3 and validated it against experimental results from ASSURE [12,13]. Subsequently, they numerically analyzed the effects of the collision angle and impact position on head injury. Rattanagraikanakorn et al. [14] developed a multibody system model of DJI Phantom III UAV impacting the 50th Hybrid III dummy model (filename: d_hyb350el_Q) in MADYMO and validated the model by experimental data. As the method was validated, the head and neck injury risk were further investigated using a biomechanical human body model (filename: h_occ50fc), and evaluated based on the criteria of HIC and Nij, respectively. And, the results showed that the UAV with a mass of approximately 1.2 kg can inflict serious head injury. As a continued study [15], the difference in the head and neck responses between the Hybrid III dummy and the human body model was investigated. It was found that the HIC predicted by these two models was almost similar under horizontal impact conditions, while the result predicted by the dummy was lower than that predicted by the human body model under vertical impact conditions, with an error of about 20% at a dropping speed of 18 m/s. Additionally, the difference in the neck Nij predicted by these two models was greater than that of the head. Li et al. conducted a comparative study of head and neck injury patterns under different impact speeds, angles, and positions by collision simulations between the Y3 drone and the Hybrid III 50th percentile dummy [16].

Koh et al. investigated drone collisions with a dummy head, using an impact energy of 95 J as the AIS3+ injury threshold for the head while considering neck injury [17,18]. Wang et al. developed a simulation model of the DJI M200 drone impacting the dummy, validated its accuracy experimentally, and analyzed the effects of impact speed, angle, and position on dummy responses [19]. They concluded that the threshold for head and neck injury from the M200 drone was 7–9 m/s, with the head and battery of the drone posing the greatest injury risk. Campolettan et al. conducted dummy collision tests with DJI drones and found that the injury risk from vertical drop impacts is higher than that from flight impacts and increases with drone mass [20]. ASSURE systematically investigated the safety threats of drones to humans via collision tests and simulations by Hybrid III and THUMS models, establishing a relationship between the drone collision kinetic energy and human injury risk [13,21]. The human injury risk was analyzed based on the head peak acceleration, HIC, 3 ms acceleration, BrIC, concussion probability, upper neck force and torque. In addition, the risk of skull fracture was further analyzed based on the skull strain. Compared with the cadaver impact tests, the simulated head responses from both the Hybrid III dummy and THUMS models were low. In addition, compared to THUMS, the Hybrid III dummy model output lower head responses and higher neck responses. Stark et al. investigated the effects of collision speed, angle, and position on human injury responses in tests involving drone collisions with cadavers. They found that the injury risk curves used in automotive testing over predicted the injury risk in drone impact scenarios. Nonetheless, it is noted that the dropping speeds under the vertical drop collision tests were around 20 m/s [22].

The above studies provide valuable insights into the safety of drone collisions with humans. However, there remains a lack of methods for injury assessment and classification after drone collisions, and comparative studies on the risk of injury from drones of different masses are limited as well. The present study addresses these gaps via the following works: (1) Drop collision tests of three different configurations of light and small drones with the Hybrid III dummy are performed. (2) The effects of drop height and impact position on head and neck injury are investigated. (3) A safety grade classification method focusing on drone impacting dummy under drop collisions is proposed. (4) The safety grades for different drones are classified based on the drop height.

## 2. Materials and Methods

### 2.1. Samples

This study involves three light and small quadrotor drones: DJI Mavic Air (referred to as Air), Mavic 2 Pro (referred to as Mavic 2), and M200. These drones comprise arms, fuselage, battery, motor, and propellers. Table 1 presents their shape, weight, and dimensions.

### 2.2. Experiment Setup

**Figure 1:**
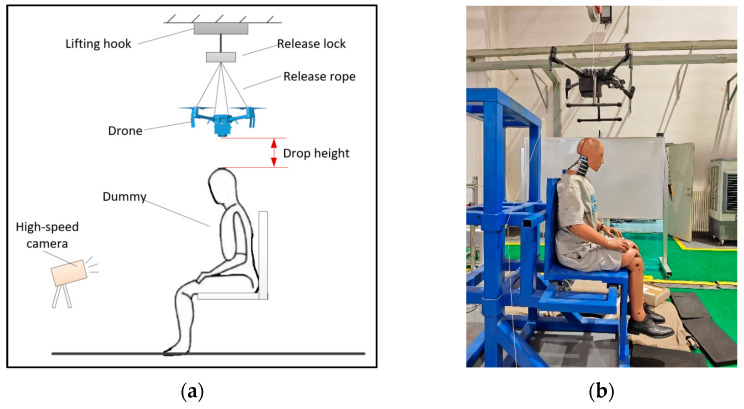
Schematic diagram and on-site photos of tests involving drone dropping and collision with dummy; (**a**) Schematic diagram of the experiment setup; (**b**) On-site photos of the setup.

The experiments were performed by releasing the drone from a specific height to impact the head of the dummy seated on a chair. The experimental setup is illustrated in Figure 1, where a lifting hook and a release lope was utilized to suspend and release the drone. Prior to each trial, the drop height was adjusted using the lifting hook, and a laser rangefinder ensured the desired height was accurately achieved. The impact position was adjusted by controlling the drone attitude through the release lock and release rope. Upon release, the drone drops freely and impacts the dummy without causing apparent attitude changes. A high-speed camera captured the attitude changes of the drone and the dummy during the experiment. The dummy, a 50th percentile Hybrid III model, was equipped with triaxial accelerometer sensors in the head, as well as triaxial force and moment sensors in the neck. The collected head acceleration and neck force were filtered by CFC1000, while the neck moment was filtered by CFC600 according to SAE J211 [23].

### 2.3. Experimental Matrix

**Figure 2:**
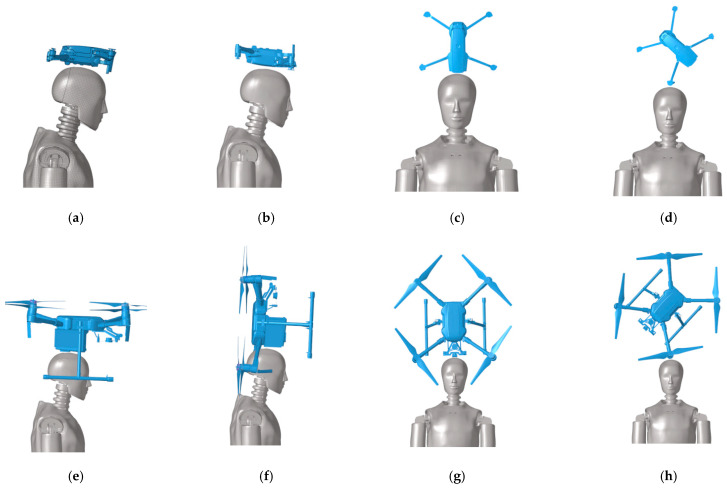
Schematic diagram of drone impacting the dummy at different positions; (**a**) Body impact, (**b**) Back impact, (**c**) Front impact, (**d**) Arm impact, (**e**) Body impact, (**f**) Tail impact, (**g**) Front impact, (**h**) Arm impact.

The experimental matrix for the drone drop tests (Table 2) was designed to investigate the effects of drop height and collision position on the Hybrid III dummy. For the vertical drop tests, Air and Mavic 2 drone models were released from initial altitudes of 1, 3, 5, 7, 9, and 11 m. Owing to its significantly higher mass, the M200 drone underwent modified safety protocols with reduced test heights of 0.1, 0.3, 0.5, 1, 3, and 5 m. As regards the collision position tests, all drones were dropped from an initial height of 5 m. Considering the different stiffness of their structural parts (Figure 2a–d), the Air and Mavic 2 drones were dropped at four typical positions of the dummy (body, back, front, and arm). Since the battery contributes remarkably to the mass of the M200 drone, collisions were tested at four typical positions (body, tail, front, and arm) to account for this factor (Figure 2e–h).

### 2.4. Head and Neck Injury Criteria

This study investigates the head and neck injury resulting from drone drops. Head injury is assessed using the head injury criterion (HIC) and the head acceleration 3 ms (head acc 3 ms), while neck injury is evaluated based on the axial force $F_{z}$, shear force $F_{x}$, and bending moment $M_{y}$. Detailed descriptions of these metrics can refer to [23]. The thresholds for each injury metric are summarized in Table 3. HIC is expressed as

(1) $$HIC={\left[(t_{2}-t_{1}){\left(\frac{1}{t_{2}-t_{1}}\int_{t_{1}}^{t_{2}}a(t)dt\right)}^{2.5}\right]}_{max}$$

where $t_{1}$ and $t_{2}$ denote the start and end times of the integration interval, respectively, while a(t) represents the resultant acceleration of the head’s center of gravity during impact (g), with commonly used time intervals of 36 ms (${HIC}_{36}$) and 15 ms (${HIC}_{15}$). The duration of the head acceleration response to drone impacts typically falls within 15 ms. Here, ${HIC}_{15}$ is utilized for the head injury analysis, with a threshold of 700.

Another widely used criterion for head injury assessment is the head acc 3 ms, which considers the cumulative time during impact where the head acceleration exceeds a certain limit, but not 3 ms. This criterion underscores the importance of limiting the acceleration duration that exceeds a certain threshold; the threshold for this criterion is 80 g.

To assess the neck injury, special attention is given to $F_{z}$, $F_{x}$, and $M_{y}$, considering the loading conditions acting on the neck under drone impact. The thresholds for $F_{z}$, $F_{x}$, and $M_{y}$ are 4000 N, 3100 N, and 57 Nm, respectively.

## 3. Results

### 3.1. Effect of Drop Height

#### 3.1.1. Experimental Results of Drones Impacting Dummy Head at Different Drop Heights

**Figure 3:**
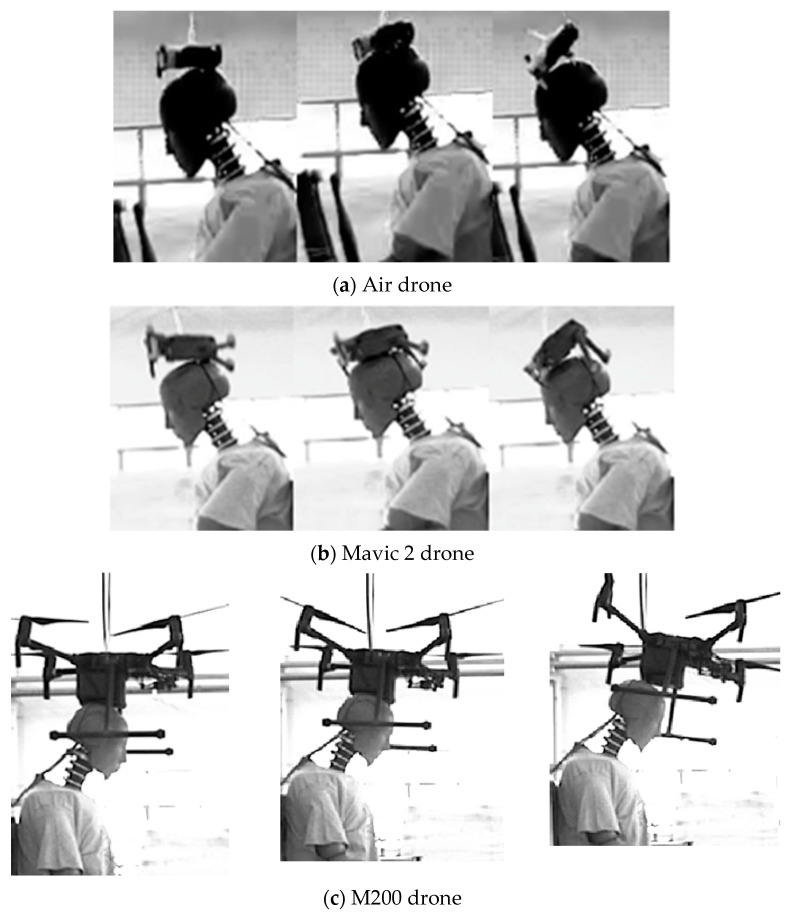
Typical responses of the dummy head after being hit by different drones.

The typical processes of the drones dropping and impacting the dummy head are depicted in Figure 3. For the Air drone, with the collisions, the dummy neck bended forward, the body of the drone underwent deformation, and its arms were broken. Subsequently, the drone rebounded and rolled due to the reaction force. Although the collision process from different heights was basically similar, the forward flexion of the dummy neck and the deformation level of the drone increased with increasing drop height. For the Mavic 2 drone, after the drone body impacted the dummy head, the dummy neck flexed forward, and the drone arms were deformed. The Mavic 2 drone, whose arms are longer compared to those of the Air drone, experienced a more significant bending moment during the collision, resulting in more pronounced bending. For the M200 drone, the battery of the drone, which is located at its bottom and occupies a significant portion of it, primarily impacted the dummy head. During collision, the dummy neck flexed forward, and the flexion was more pronounced compared to that of the other two drones.

**Figure 4:**
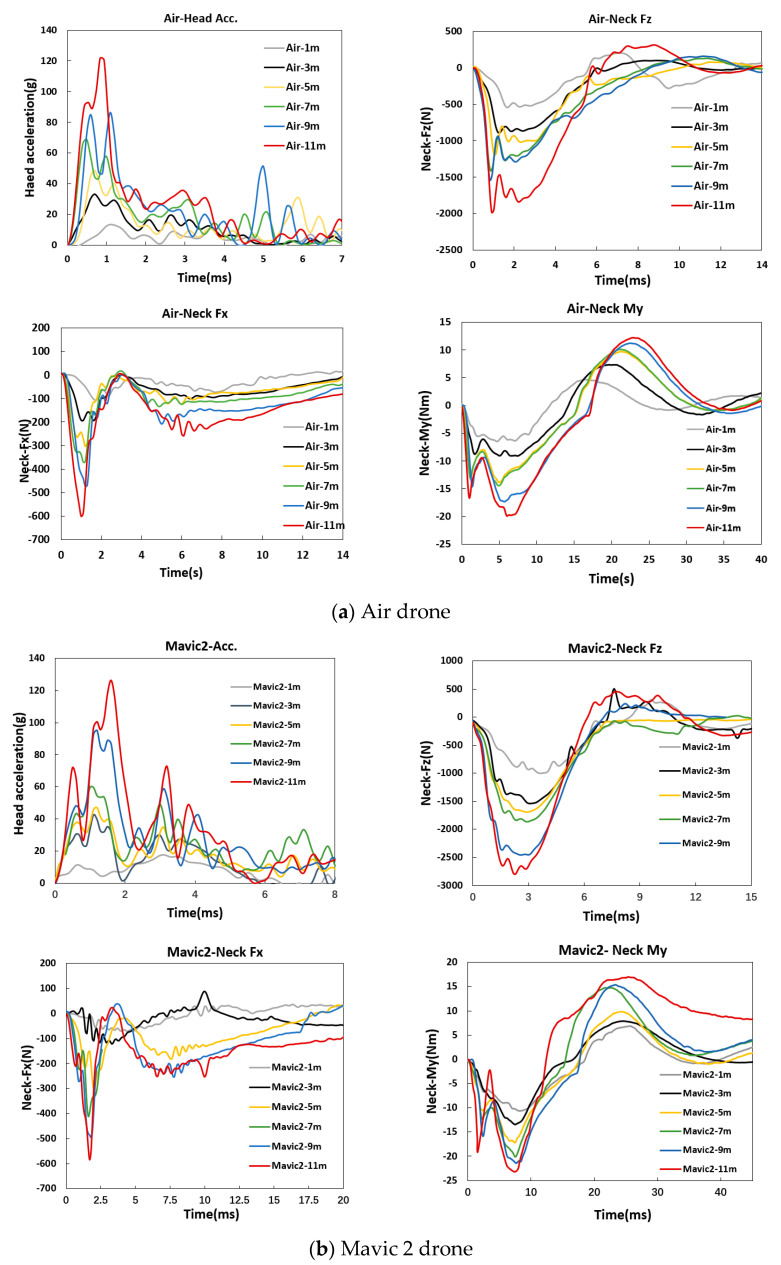

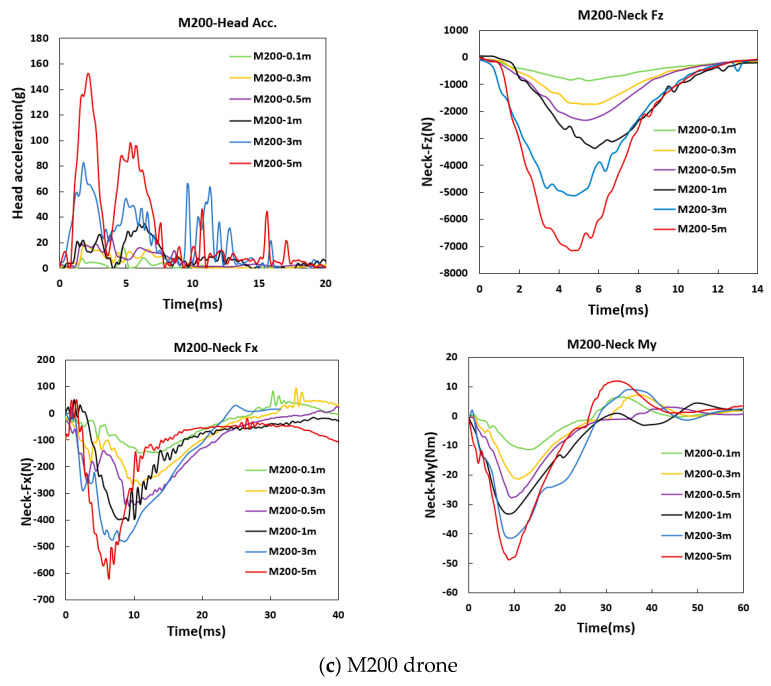
Responses of dummy head and neck after collisions with different drones from varied drop heights.

Figure 4a illustrates the head resultant acceleration, neck force, and neck-bending moment of the dummy after collision with the Air drone from different drop heights. The response curves of the dummy head and neck exhibited similar trends with time. The head acceleration curve demonstrated a rapid increase, peaking at 1 ms, followed by a gradual decrease. The peak acceleration value increased progressively with increasing drop height. As regards the neck response, the change in shear force was more pronounced than that of the axial force, quickly falling to nearly zero after peaking. Both the axial force and bending moment of the neck decreased after reaching their peak values, persisting for some time. Initially, the neck-bending moment was negative, causing the dummy neck to extend backward. As the collision progressed, the drone compressed the dummy head, with the bending moment transitioning gradually from negative to positive, and the neck shifting gradually from extension to forward flexion. After the collision, the neck-bending moment decreased gradually to zero.

Figure 4b depicts the head resultant acceleration, neck force, and bending moment curves after collision with the Mavic 2 drone from different drop heights. Overall, a gradual increase in the head and neck responses with increasing drop height was observed. The Mavic 2 drone exhibited a similar head acceleration pattern to that of the Air drone, while its acceleration duration was significantly longer. As regards the neck axial force, unlike the Air drone, the Mavic 2 drone curve exhibited a smoother profile without significant peaks at the different heights. This smoother curve can be attributed to the larger gap between the outer shell and the internal structure of the Mavic 2 drone. At heights lower than 9 m, where the gap was not fully eliminated upon collision, deformation primarily occurred in the outer shell, resulting in the absence of significant peaks or fluctuations. Nevertheless, at the drop height of 11 m, fluctuations in the neck axial force were observed, possibly due to the full compression of the gap between the outer shell and the internal structure; still, they were less pronounced than those of the Air drone. Although the peak neck shear force and bending moment values were similar to those of the Air drone, the response duration was slightly greater for the Mavic 2 drone.

Figure 4c illustrates the head resultant acceleration, neck force, and neck-bending moment of the dummy after collision with the M200 drone from different drop heights. The maximum head acceleration, neck axial force, neck shear force, and neck-bending moment values increased with increasing drop height. Compared to the Air and the Mavic 2 drones, the peak values of the M200 drone were larger at the same drop height. All three drones exhibited a similar head acceleration pattern, with distinct double peaks at the initial collision moment, which were followed by oscillations that became wider with increasing drone mass. Unlike the Air drone that exhibited a peak at the initial collision moment, the neck axial force of the M200 drone was more similar to that of the Mavic 2 drone, since there was no significant stiffness change at the collision site. The trend of the neck shear force was similar among all three drones. As regards the neck-bending moment, there was a certain delay compared to the neck forces (axial and shear forces). The neck-bending moment reached its peak when the neck force dropped to zero. This behavior was consistent with that of the Air and Mavic 2 drones; however, the neck-bending moment of the M200 drone did not exhibit significant double peaks. This difference may be due to the collision of the M200 drone with the dummy, which not only caused neck deformation but also propelled the entire dummy forward due to its larger mass, thereby canceling out the oscillations of the neck-bending moment.

#### 3.1.2. Analysis of Results

**Figure 5:**
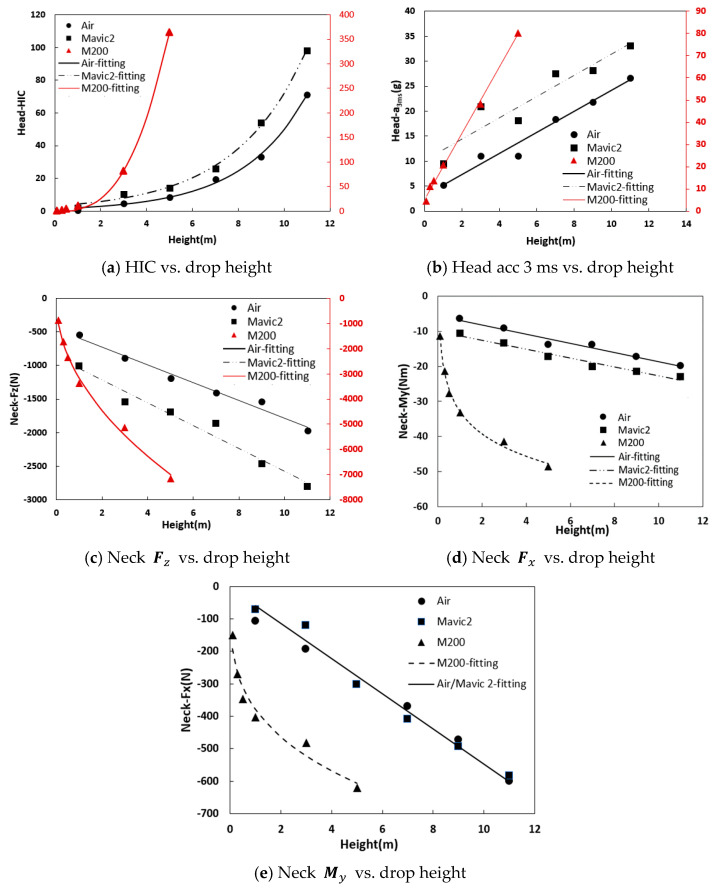
Variation trends of head and neck injury metrics with drop height.

The head and neck injury metrics were calculated according to the criteria outlined in Section 2.4. The relation between these metrics (HIC, head acc 3 ms, $F_{z}$, $F_{x}$, and $M_{y}$) and the drop height was established, as illustrated in Figure 5. The head and neck injury metrics for all three drones increased with increasing drop height. In addition, for the same height, the greater the mass of the drone, the higher the values of the injury metrics.

The trends of the injury metrics with respect to the drop height were not identical. The head HIC for all three drones exhibited an exponential increase with height, while head acc 3 ms increased linearly. The increase rate of HIC and head acc 3 ms was higher for the M200 drone compared to the other two drones. The neck injury metrics ($F_{z}$, $F_{x}$, and $M_{y}$) for the Air and Mavic 2 drones exhibited a linear increase with increasing drop height; on the other hand, the neck axial force ($F_{z}$) and shear force ($F_{x}$) for the M200 drone increased as a power function, while the neck-bending moment ($M_{y}$) followed a logarithmic growth relationship.

The relations between the injury metrics (HIC, head acc 3 ms, $F_{z}$, $F_{x}$, and $M_{y}$) and the drop height for the three typical drones were fitted, and the respective equations are provided below. It should be noted that the parameters on both sides of the equation were not normalized before fitting to become dimensionless, since the chosen expression style is more intuitive to reflect the relationship between each injury metric and drop height. Based on these equations, the specific values of the head as well as neck injury metrics for the three drones falling from different drop heights can be directly predicted.

Air drone,
  (2) $$\left\{\begin{array}{c} HIC={1.444e}^{0.354(Height)}\;\;\;\;\;\;\;\;\;\;\;\;\;\;\;\\ a_{3ms}=2.102\left(Height\right)+3.110\;\;\;\;\\ Fz=-132.96(Height)-458.03\\ Fx=-54.204(Height)-6.669\;\;\\ My=-1.311\left(Height\right)-5.564\;\;\; \end{array}\right.$$
  Mavic 2 drone,
  (3) $$\left\{\begin{array}{c} HIC={3.013e}^{0.317(Height)}\;\;\;\;\;\;\;\;\;\;\;\;\;\;\;\\ a_{3ms}=2.138\left(Height\right)+10.098\;\;\;\;\\ Fz=-132.96(Height)-458.03\\ Fx=-54.204(Height)-6.669\;\;\\ My=-1.311\left(Height\right)-5.564\;\;\; \end{array}\right.$$
  M200 drone,
  (4) $$\left\{\begin{array}{c} HIC={6.619e}^{0.793(Height)}\;\;\;\;\;\;\;\;\;\;\;\;\;\;\;\;\;\;\;\\ a_{3ms}=14.872\left(Height\right)+5.255\;\;\;\;\;\\ Fz=-3160{\left(Height\right)}^{0.495}\;\;\;\;\;\;\;\;\;\;\;\;\;\;\;\;\;\;\\ Fx=-378.5{\left(Height\right)}^{0.293}\;\;\;\;\;\;\;\;\;\;\;\;\;\;\;\;\;\\ My=-9.219ln\left(Height\right)-32.905\;\;\; \end{array}\right.$$

### 3.2. Effect of Collision Position

#### 3.2.1. Experimental Results of Different Positions of Drones Impacting Dummy Head

**Figure 6:**
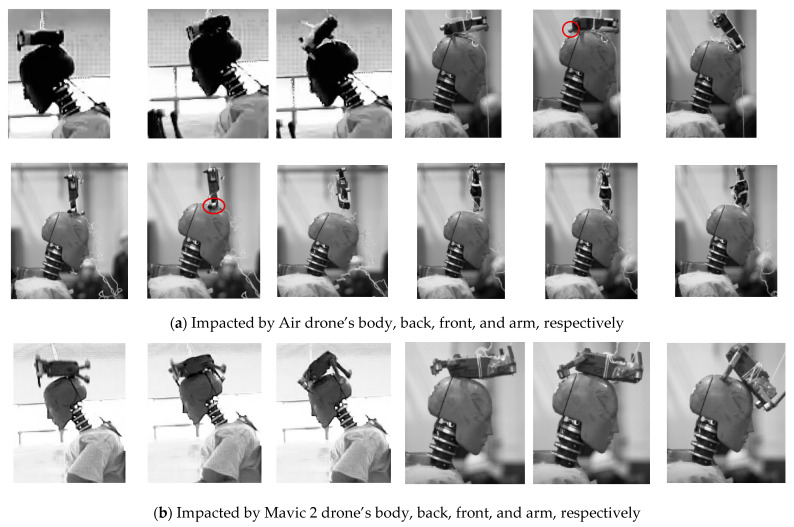

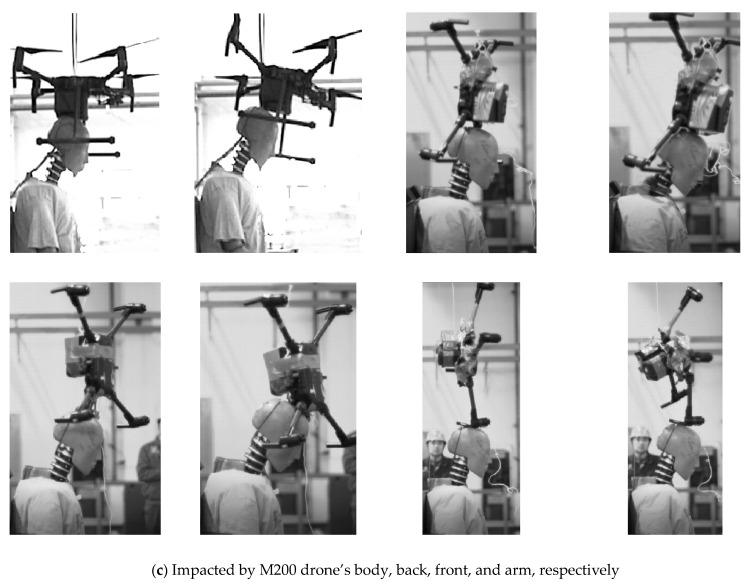
Dummy head/neck trajectories under impact from different positions of the drones Air, Marvic 2, and M200.

The different positions of the Air drone impacting the dummy are illustrated in Figure 6a. When the body and back of the Air drone impacted the dummy head, no significant deformation occurred due to the higher stiffness and larger contact area of these parts. However, the Air drone arms exhibited noticeable bending during the collisions, and the dummy head rolled off after impact. When the Air drone front collided with the dummy head, the front experienced slight deformation and the arms bent, causing the drone to bounce off. When the Air drone arms collided with the dummy head, they gradually bent until they collapsed, causing the drone to slide off the head. Throughout these collisions, the dummy neck exhibited slight forward flexion.

Figure 6b illustrates the changes in the dummy posture after collision with different positions of the Mavic 2 drone. When the Mavic 2 body and back collided with the dummy head, the drone arms experienced significant deformation and bending due to inertial forces, causing it to roll forward and fall off the dummy head. In the collisions between the Mavic 2 front and the dummy head, the elasticity of the drone front caused it to bounce off. During impact with the Mavic 2 arms, the motor bracket that mounts the rotors deformed first, which was followed by overall bending of the arms, causing the drone to deviate and fall off. The dummy neck exhibited certain forward flexion under all impact scenarios.

Due to the structural differences between the M200 drone and the other two drones, impact tests were conducted with four different positions of the drone: body, front, tail, and arms. Figure 6c illustrates the responses of the dummy to the collision from these different positions. When the M200 body impacted the dummy head, the battery beneath the body collided with the head, causing significant forward flexion of the dummy neck, while the drone slid off the front of the dummy head. In the M200 tail collision scenario, both the tail and the battery struck the dummy head, resulting in a posture change similar to that observed under the body collision. During the M200 front collisions, the camera mounted on the drone front collided with the dummy head, gradually deforming and breaking, before sliding off the top of the dummy head. During the M200 arm collisions, the arms experienced severe bending damage from the strong impact and slid off the side of the dummy head, causing forward flexion of the dummy neck. Overall, due to the heavier mass of the M200 drone, the dummy neck exhibited more pronounced forward flexion compared to that under the other two drones.

**Figure 7:**
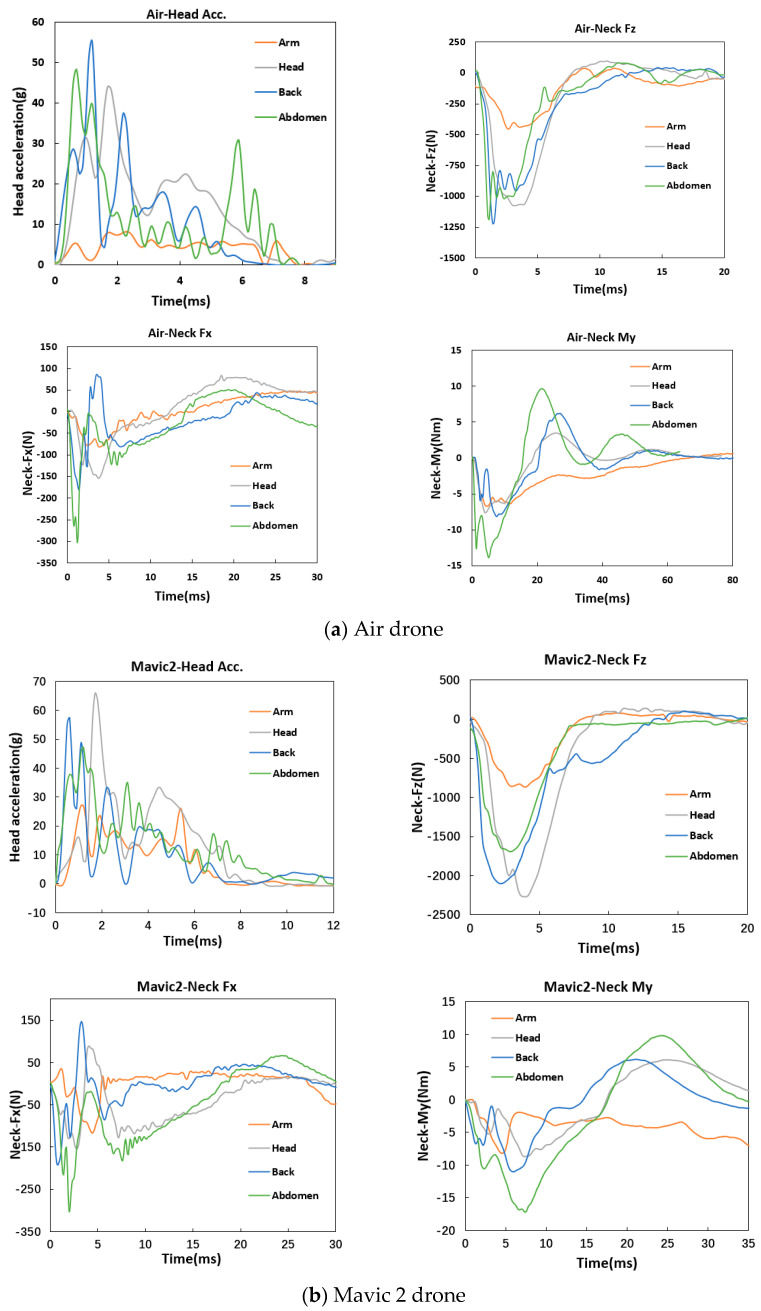

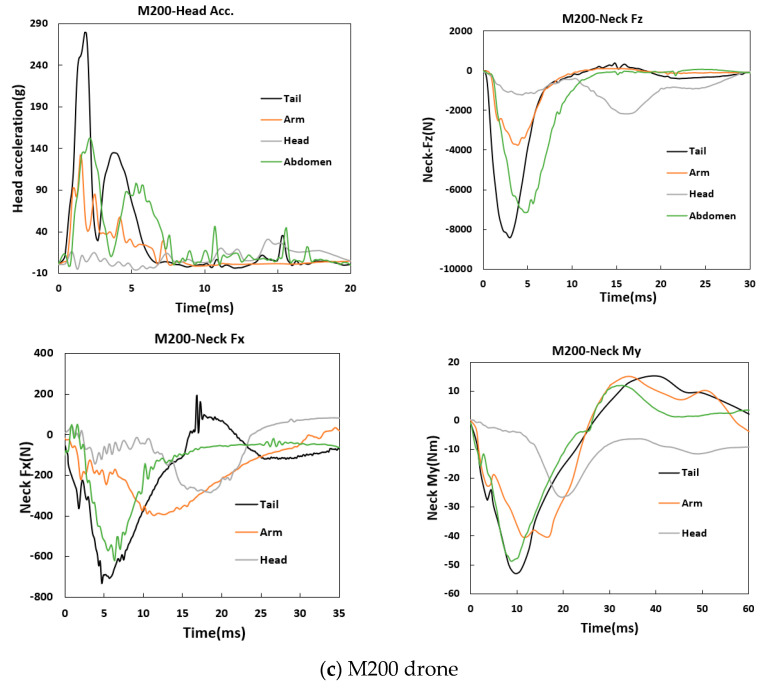
Dynamic responses of the dummy head and neck with impact from different positions of the Air, Mavic 2, and M200 drones.

The dynamic responses of the dummy head and neck to collisions by distinct positions of the Air drone are shown in Figure 7a. Overall, the collisions with the Air drone arms resulted in significantly lower responses compared to those with the other three positions, which was likely due to the fact that the large deformation of the arms absorbed more energy. The maximum head acceleration value occurred under Air drone back collisions, which was followed by slightly lower values under the Air drone body and front collisions. The acceleration curves from back and body impacts displayed distinct multi-peak oscillations, likely due to the drone rolling on the dummy head during impact. The neck axial force curves for the Air drone back and front collisions increased rapidly to a peak and then decreased quickly, eventually dropping to zero. This is due to the fact that the Air drone back and body collisions involved initial collisions with the outer shell, causing deformation and compression of the internal gaps. Then, the elastic deformation of the outer shell gradually recovered until the drone separated from the dummy head. The highest values of the neck shear force and bending moment were observed for the Air drone body collisions, followed by the back and front collisions, with the lowest values measured under the arm collisions. The shear force and bending moment curves for the Air drone arm, front, and body collisions exhibited clear oscillations with multiple peaks. In contrast, the shear force and bending moment curves for the arm collisions decreased gradually to zero after reaching their peaks, without exhibiting multi-peak fluctuations.

Figure 7b presents the dynamic responses of the dummy head and neck to collisions by distinct positions of the Mavic 2 drone. Due to the structural similarities between the Mavic 2 and Air drones, the head and neck response patterns were also similar. As regards the Air drone, the arm collisions resulted in the weakest head and neck responses. More specifically, the peak head acceleration values for the Mavic 2 front, back, and body collisions were similar and significantly higher than those for the arm collisions. The trends of the acceleration curves showed that, similar to the Air drone, the collisions from the Mavic 2 back and front positions caused greater oscillations to the dummy head acceleration compared to those from other positions. The peak neck axial force patterns from the different impact positions were consistent with those for the acceleration. The highest peak occurred from the Mavic 2 front collisions, followed by slightly lower peaks from the Mavic 2 back and body collisions, with the arm collisions resulting in the lowest peaks. The peak neck shear force and bending moment values that occurred from Mavic 2 collisions and their overall trends were similar to those observed with the Air drone.

Figure 7c shows the dynamic responses of the dummy head and neck to collisions by distinct positions of the M200 drone. The highest peaks in the head and neck occurred from M200 tail collisions, while the lowest ones occurred from M200 front collisions. In particular, as regards the head acceleration, the M200 tail collisions produced the highest peaks, followed by those from body and arm collisions, with the head collisions resulting in significantly lower peaks. The acceleration curves from M200 tail and body collisions were similar, both exhibiting two distinct peaks. This is attributed to the M200 battery, which occupies a significant portion of the drone, and impacted the dummy head during both body and tail collisions. The big stiffness of the battery resulted in significant impact forces, causing an initial acceleration peak as the dummy head moved downward under impact and a secondary peak during compression. Under the M200 front collisions, the camera contacted the dummy head first, causing gradual compression in neck axial force. For the low stiffness of the camera, the head acceleration was the lowest among all impact cases. Under arm collisions, the acceleration initially increased and then decreased as the arms bent. The patterns of the peak neck axial force values were similar to those for the head acceleration, with the highest peak occurring under M200 tail collisions and the lowest under head collisions. The curves for the tail, body, and arm collisions exhibited a similar trend, i.e., increasing first and then decreasing to zero. Moreover, the neck overall lower stiffness indicated that it did not exhibit the two-peak pattern observed in the head acceleration. During the M200 front collisions, the axial force was initially low and increased as the camera underwent gradual compression. As to the neck shear force and bending moment, the highest peaks occurred under M200 tail collisions, with the lowest under head collisions. The overall trends of the curves from the M200 body, tail, and arm collisions were similar, with peaks occurring later under arm collisions than under tail and body collisions, likely due to the deformable characteristics of the arms during the collision. The shear force and bending moment curves from head collisions differed from those from the other positions, exhibiting a significant increase of about 10 ms after collision, likely due to the increased stiffness resulting from the compression of the camera.

#### 3.2.2. Results Analysis

**Figure 8:**
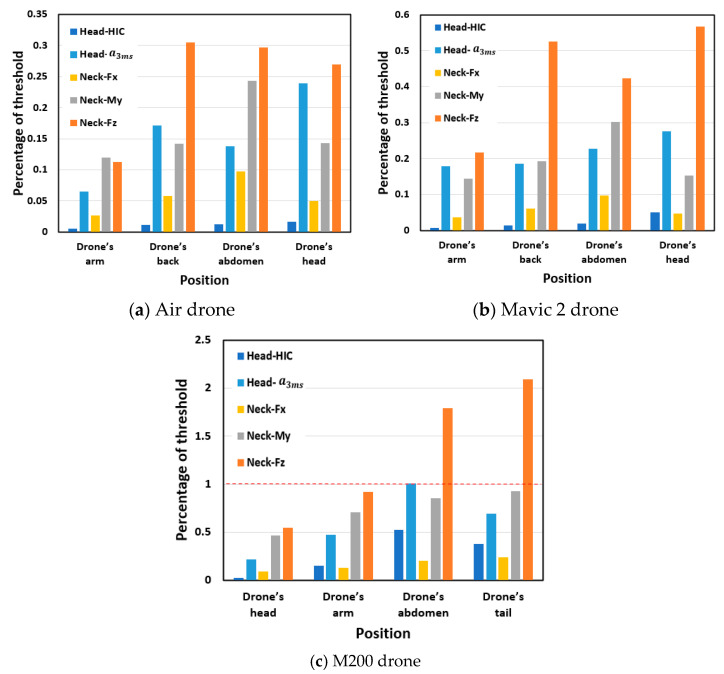
Dummy’s percentage of threshold induced by the impact of drones’ different positions.

The dummy head and neck injury metrics, calculated according to the criteria in Section 2.4, were normalized by dividing each metric by its respective threshold value, referred to as the “percentage of threshold”. The results of dummy head and neck responses induced by the collision of drones’ different positions are demonstrated in Figure 8. As regards the Air and Mavic 2 drones, the dummy head and neck peak values from drone’s body, back, and front collisions were generally high and comparable. In contrast, the results from the drone’s arm collisions were significantly lower. Due to its greater overall mass, the Mavic 2 drone produced higher dummy head and neck responses under all impact scenarios compared to the Air drone. As regards the M200 drone, the dummy head and neck responses from drone’s body and tail collisions were significantly higher than those from drone’s front and arm collisions. The injury metrics for the M200 drone under all impact scenarios were significantly higher than those for the Mavic 2 and Air drones. Notably, the neck axial force metrics from the drone’s body and tail collisions exceeded the threshold values when the M200 drone was dropped from a height of 5 m.

## 4. Safety Grade Classification of Drone Collision with Dummy

### 4.1. Safety Grade Classification Method Development

**Figure 9:**
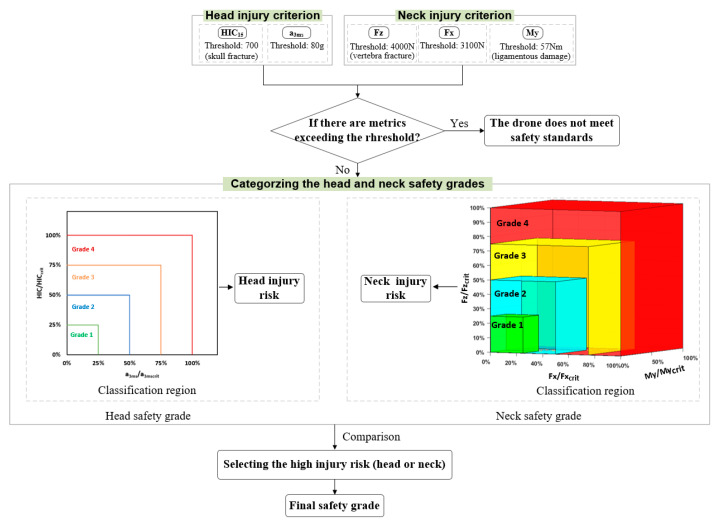
Procedure for determining the safety grades.

Referring to the basic idea of Austen et al. [29], this study presents a procedure for determining the safety grades of drone collision with dummy. The procedure is outlined in Figure 9. To evaluate head and neck injury from drone collisions, various metrics are extracted and compared against threshold values. If a metric exceeds its threshold, this indicates that the drone has caused injury and fails to meet safety standards. Conversely, if none of the metrics surpass their thresholds, the safety of head and neck injury is classified. Subsequently, the injury risks for the head and neck are compared, and the higher risk is adopted as the final collision safety grade of the drone.

Specifically, relying on a single metric may not offer a comprehensive evaluation; thus, this section adopts the typical head and neck injury metrics (Section 2.4) to perform a classification based on the ratio of the injury metrics to their thresholds (percentage of threshold). As mentioned above, the head injury metrics, including the HIC and head acc at 3 ms, form a two-dimensional distribution. The neck injury metrics, including the axial force $F_{z}$, shear force $F_{x}$, and bending moment $M_{y}$, form a three-dimensional distribution. The values below the threshold were divided into four uniform safety grades, with Grade 1 representing the lowest injury risk (highest safety grade) and Grade 4 the highest injury risk (lowest safety grade).

### 4.2. Safety Grade Classification for the Three Typical Drones

**Figure 10:**
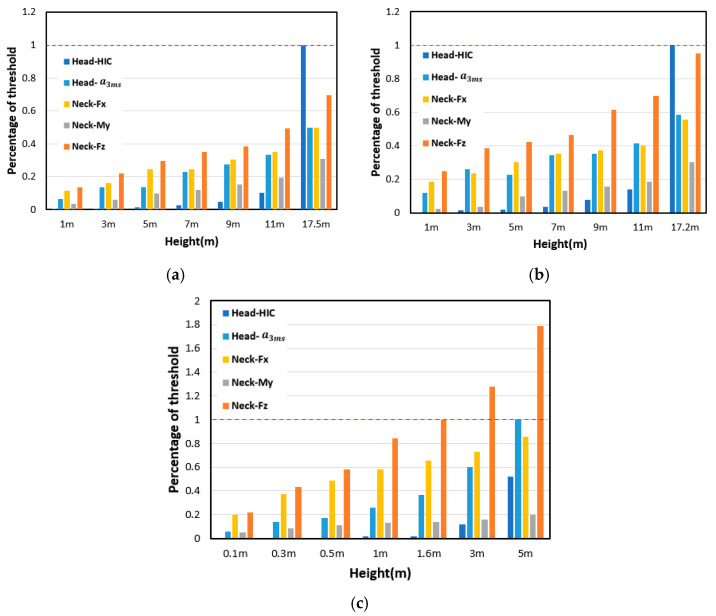
Percentage of threshold for the three typical drones under different drop heights; (**a**) Air drone, (**b**) Mavic 2 drone, (**c**) M200 drone.

To determine the safety grades associated with the drop heights of the three typical drones, the head and neck injury metrics (HIC, head acc 3 ms, $F_{z}$, $F_{x}$, and $M_{y}$) were calculated and compared with the corresponding threshold values under different drop heights (Figure 10). It was found that, as regards the Air and Mavic 2 drones, the head HIC exceeded the threshold value when the drop height exceeded 17.5 m (Air) and 17.2 m (Mavic 2), while, as regards the M200 drone, the neck axial force reached the threshold value at a height of 1.6 m.

**Figure 11:**
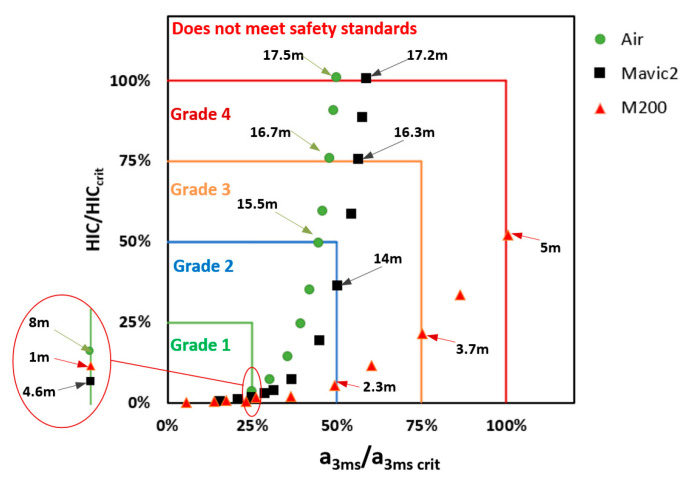
Classification of head safety grades for the three typical drones falling from different drop heights.

Based on the percentage of threshold of the injury metrics and following the safety grade classification method established in Section 4.1, the head and neck injury safety grades induced by the three typical drones falling from different drop heights were determined. The classification results for the head are depicted in Figure 11. For the Air and Mavic 2 drones, the head acc 3 ms is the primary factor affecting head injury up to Grade 2, after which HIC becomes dominant. As for the M200 drone, the head acc 3 ms metric is more likely to reach the threshold value compared to HIC, making it the primary factor in head injury classification.

**Figure 12:**
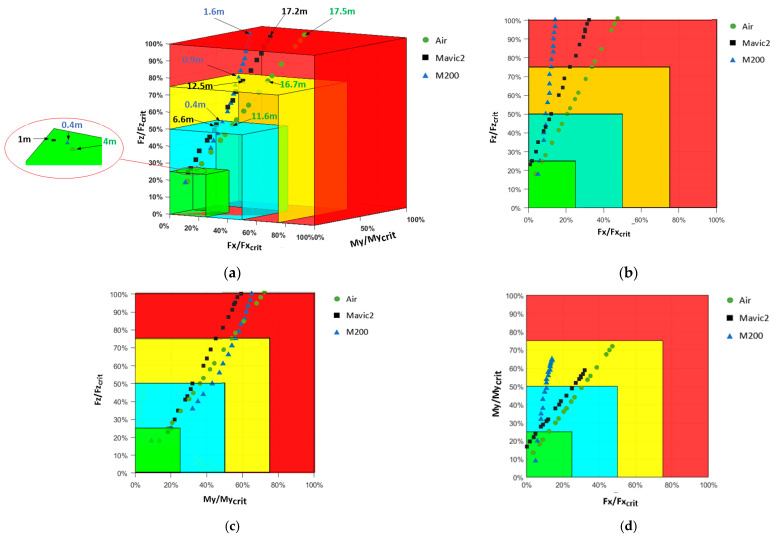
Classification of neck safety grades for the three typical drones falling from different drop heights; (**a**) Neck safety grade classification, (**b**) Neck shear force vs. axial force, (**c**) Neck-bending moment–axial force, (**d**) Neck shear force vs. bending moment.

The classification results for the neck are exhibited in Figure 12a. The primary factor affecting the neck injury classification results is the neck axial force. This is consistent with scenarios where drones dropped vertically and impacted the dummy head. Notably, for the M200 drone, the neck axial force is the dominant factor due to the drone’s larger mass, which causes a substantial increase in axial force upon impact.

To further analyze the effects of metrics such as the neck axial force, shear force, and bending moment on neck injury, the neck injury metrics were projected onto two coordinate planes. Figure 12b shows that the neck axial force increase rapidly, while the neck shear force change minimally with increasing drop height. In general, the larger the mass of the drone, the more pronounced this phenomenon, with the neck axial force being the primary factor leading to injury. As regards the neck axial force and bending moment (Figure 12c), both begin to increase significantly with increasing drop height, with the neck axial force exhibiting a greater trend, making it the primary factor leading to injury. Figure 12d presents the relationship between neck-bending moment and shear force, indicating that the neck-bending moment increases more significantly with increasing drop height, especially for larger drones, with the neck-bending moment being the primary factor leading to injury compared with the neck shear force.

Based on the method proposed in this study for classifying the safety grades for vertical drop collisions of drones, the safety grades for the three typical drones falling from different drop heights are summarized in Table 4. For the Air drone, the grades are as follows: Grade 1 from 0 to 4 m, Grade 2 from 4 to 11.6 m, Grade 3 from 11.6 to 16.7 m, Grade 4 from 16.7 to17.5 m, and it fails to meet safety standards beyond 17.5 m. For the Mavic 2 drone, the grades are: Grade 1 from 0 to 1 m, Grade 2 from 1 to 6.6 m, Grade 3 from 6.6 to 12.5 m, Grade 4 from 12.5 to 17.2 m, and it fails to meet safety standards beyond 17.2 m. For the M200 drone, the grades are: Grade 1 from 0 to 0.1 m, Grade 2 from 0.1 to 0.4 m, Grade 3 from 0.4 to 0.9 m, Grade 4 from 0.9 to 1.6 m, and it fails to meet safety standards beyond 1.6 m.

## 5. Discussion and Conclusions

This study conducted collision tests on the Hybrid III dummy with three typical drones falling from distinct heights and orientations to simulate drones impacting dummy head and analyzed the effects of drone type, drop height, and drone impact positions on head and neck injury, respectively. A method was proposed to classify the safety grades of drone collisions with the dummy head under vertical drop and the drop heights of the three drones were categorized. Limitations still exist for this study:

(1) This study is experimental in nature. While cadaver tests are considered the most accurate method for representing human responses to collisions, ethical and legal constraints make it challenging to obtain cadaver specimens for such tests. As an alternative, we conducted the drone collision tests using the crash dummy and investigated the head and neck injury risk. A limitation is that the dummy cannot be equivalent to the true human, and certain differences exist between the dummy and the true human. For example, the Hybrid III dummy’s neck exhibits high stiffness and lacks biofidelity compared to a human neck [30]. This difference can lead to deviations between the acquired head and neck dynamic responses and those of a human. However, the development of the dummy is also based on some biomechanical data, and it can be repeatedly used to conducted tests according to certain procedures, thus it is widely used in the automotive and aviation industries. While the head and neck mechanical responses measured from Hybrid III dummy-based drone collision tests may differ from those of a human in specific collision scenarios, the relative law of head and neck mechanical responses induced by different collision conditions and locations should be accurate, and can provide valuable guidance for drone safety design. It is noted that the exact corresponding relationship between the measurements obtained for a drone hit of a Hybrid III dummy to a drone hit of a true human need to be further explored in future.
(2) This study presents a method for classifying the safety grades of small drones. A limitation is that the safety grades were obtained based on the head and neck responses from Hybrid III dummy instead of a human. Due to the difference existing between the dummy and a human, the safety levels assigned to small drones in this study may not fully capture the actual safety levels in collisions involving humans. Nevertheless, the method for classifying small drone safety levels is effective and the classification framework can be also appliable to humans. It is noted that the classification results based on the human results (once the data were obtained) may be varied when compared to the results based on the dummy results, which remains to be addressed in our follow-up work.
(3) According to Klevien [31], brain tissue injury is more sensitive to rotational motion relative to linear motion. This is mainly due to the fact that the bulk modulus of brain tissue is about five to six orders of magnitude higher than its shear modulus, and the brain tissue strain induced by rotational loading is more sensitive when a head collision occurs. Head linear dynamics (e.g., linear acceleration, impact force, and HIC) primarily correlate with skull fractures and are insufficient for evaluating brain injuries resulting from rotational motion. Such rotational injuries, which constitute the majority of brain injuries, include concussion, diffuse axonal injury, contusion (in absence of skull fracture), subdural hematoma, and intra-cerebral hematoma. This is a limitation of this study because no angular kinematics were collected, although head rotation is small under vertical drop. Next step, head rotational dynamics under various collision conditions (e.g., frontal, rear, and oblique collisions) between UAVs and human heads should be collected and analyzed to investigate brain injury.

In a word, the main concluding remarks are summarized as follows:

(1) The variation law of the injury metrics of the dummy head and neck with drop height was obtained. As the drop height of the drones increases, the HIC and head acc. 3 ms metrics exhibited exponential and linear growth, respectively, with increasing drop height for all drones. However, the M200 drone exhibited a notably faster increase rate in the HIC and head acc 3 ms upon impact than the Air and Mavic 2 drones. As for neck injury, the three metrics ($F_{z}$, $F_{x}$, and $M_{y}$) for the Air and Mavic 2 drones increased linearly with increasing drop height. In contrast, the M200 drone presented a power function relation for $F_{z}$ and $F_{x}$, and a logarithmic relationship for $M_{y}$.
(2) The impact positions of the drone also affect the dummy’s head and neck responses. For the Air and Mavic 2 drones, the head and neck responses from drone’s body, back, and front collisions were similar, while those from drone’s arm collisions were significantly lower. In the case of the M200 drone, the head and neck responses from the drone’s body and tail collisions were significantly higher than those from the drone’s front and arm collisions. In particular, when the M200 drone’s body or tail impacted the dummy vertically from a height of 5 m, the neck axial force exceeded the safety threshold.
(3) Four safety grades were classified according to the injury metrics of the dummy vertically impacting the drones, and the drop height ranges corresponding to the different safety grades were further determined.

Future research could explore more diverse collision scenarios (e.g., frontal, rear, and oblique collisions) and incorporate a wider range of drone types in collision experiments. Furthermore, highly biofidelic human finite element models (e.g., THUMS, GHBMC) could be implemented for more comprehensive simulation analyses of drone–human collisions, enabling the acquisition of more realistic mechanical responses across various human body regions.

## Figures and Tables

**Table 1:** Characteristics of the drone models.

Model	Shape	Mass (kg)	Size (mm)	
			Unfolded (L × W × H)	Folded (L × W × H)
Air	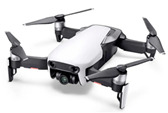	0.43	168 × 184 × 64	168 × 83 × 49
Mavic 2	** 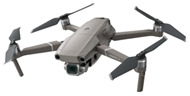 **	0.907	322 × 242 × 84	214 × 91 × 84
M200	** 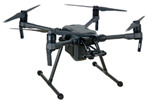 **	4.53	887 × 880 × 378	716 × 220 × 236

**Table 2:** Experimental matrix for the drone collision with dummy tests.

Drone Model	Height (m)	Impact Position
Air	1, 3, 7, 9, 11	Body (Figure 2a)
	5	Body (Figure 2a), Back (Figure 2b), Front (Figure 2c), Arm (Figure 2d)
Mavic 2	1, 3, 7, 9, 11	Body (Figure 2a)
	5	Body (Figure 2a), Back (Figure 2b), Front (Figure 2c), Arm (Figure 2d)
M200	0.1, 0.3, 0.5, 1, 3	Body (Figure 2e)
	5	Body (Figure 2e), Tail (Figure 2f), Front (Figure 2g), Arm (Figure 2h)

**Table 3:** Thresholds for the head and neck injury metrics.

Region	Types of Injury	Metric	Threshold (FMVSS 208 [24]; ECE R94 [25]; Mertz et al., 1996, 1978 [26,27]; Mertz and Patrick 1971 [28])
Head	Skull fracture	*HIC*_15_	700
	—	*a*_3ms_	80 g
Neck	Cervical vertebra fracture	*F*z (compression)	4000 N
	—	*F*x	3100 N
	Ligamentous injury	*M*y (tension)	57 Nm

**Table 4:** Drone collision safety grades based on drop height.

Drone	Grade 1	Grade 2	Grade 3	Grade 4
Air	0 ≤ h < 4 m	4 m ≤ h < 11.6 m	11.6 m ≤ h < 16.7 m	16.7 m ≤ h < 17.5 m
Mavic 2	0 ≤ h < 1 m	1 m ≤ h < 6.6 m	6.6 m ≤ h < 12.5 m	12.5 m ≤ h < 17.2 m
M200	0 ≤ h < 0.1 m	0.1 m ≤ h < 0.4 m	0.4 m ≤ h < 0.9 m	0.9 m ≤ h < 1.6 m

## Data Availability

Data are available upon request.
